# The Role of TRPC6 in the Neuroprotection of Calycosin Against Cerebral Ischemic Injury

**DOI:** 10.1038/s41598-017-03404-6

**Published:** 2017-06-08

**Authors:** Chao Guo, Yongyuan Ma, Shanbo Ma, Fei Mu, Jiao Deng, Jialin Duan, Lize Xiong, Ying Yin, Yanhua Wang, Miaomaio Xi, Aidong Wen

**Affiliations:** 1 Department of Pharmacy, Xijing Hospital, Fourth Military Medical University, Xi’an, 710032 P.R. China; 2 Department of Anesthesiology, Xijing Hospital, Fourth Military Medical University, Xi’an, 710032 PR China

## Abstract

Our previous studies have provided evidences that calycosin can protect the brain from ischemia/reperfusion injury, but its mechanisms is not fully understand. Transient receptor potential canonical 6 (TRPC6) has a critical role in promoting neuronal survival against cerebral ischemic injury. The aim of the present study is to test whether calycosin protects against cerebral ischemic injury through TRPC6-CREB pathway. *In vivo*, rats were subjected to transient middle cerebral artery occlusion (MCAO) for 2 h and then treated with different doses of calycosin at the onset of reperfusion. *In vitro*, primary cultured neurons were treated by calycosin, then exposed to 2 h oxygen glucose deprivation (OGD) followed by 24 h reoxygenation. Our results showed that treatment with calycosin protected against ischemia-induced damages by increasing TRPC6 and P-CREB expression and inhibiting calpain activation. The neuroprotection effect of calycosin was diminished by inhibition or knockdown of TRPC6 and CREB. These findings indicated that the potential neuroprotection mechanism of calycosin was involved with TRPC6-CREB pathway.

## Introduction

Stroke is a major cause of morbidity and disability in industrialized countries^1^. The overall costs of care for stroke patients will account for 6.2% of the total burden of illness by 2020. Brain injury after focal cerebral ischemia is the most common cause of stroke. Recombinant tissue plasminogen activator (r-tPA) is the only FDA approved therapy for acute ischemic stroke. However, the limited time frame for r-tPA administration and fear of hemorrhagic side effects severely restrict its clinical application^2, 3^. The need for developing an effective and safe treatment for acute stroke remains urgent. The pathogenesis of ischemic stroke is not fully understood; several underlying mechanisms have emerged such as excitotoxicity^4^, oxidative stress^5^, and calcium overload^6^, inflammation and apoptosis^7, 8^. Of these, calcium overload is regarded as the final common pathway of the mechanisms leading to neuronal death^9, 10^. Cerebral ischemia can cause the over-activation of glutamate receptors because of increased glutamate release and trigger intracellular Ca^2+^ influx. Much Ca^2+^ internal flow can activate a number of enzymes that damage cell structures, induce free radical generation and ultimately result in oxidative stress and neuron apoptosis^11^. Therefore, Ca^2+^ overload remains the central focus. It has reported in recent years that Ca^2+^ overload causes the calcium-dependent protease calpain activation^12^. The activation of calpain then leads to proteolysis of transient receptor potential canonical (subtype) 6 (TRPC6) channels. The TRPC channels are Ca^2+^-permeable, nonselective cation channels that are expressed in many cell types including neuron. Based on their amino acid sequences and functional similarities, the TRPC family can be divided into 4 groups: TRPC1, TRPC2, TRPC4/5, and TRPC3/6/7. Studies have demonstrated that TRPC6 channel has a critical role in promoting neuronal survival against cerebral ischemic injury^13–15^. Sossin *et al*. reported^16^ that TRPC6 channel regulates intracellular calcium homeostasis and consequently activates calcium/calmodulin-dependent protein kinase (CaMK) and cAMP-response element binding protein (CREB) signaling cascades to promote neuron survival. However, activation of calpain leads to TRPC6 degradation and contributes to neuronal damage in cerebral ischemia. Therefore, maintaining CREB activity by blocking calpain proteolysis of TRPC6 to preserve neuronal survival might be a new therapeutic strategy against cerebral ischemic stroke.

Calycosin, a major isoflavonoid in *Radix Astragali Mongolici*, was reported to exhibit anti-oxidative, anti-inflammatiry, tumor suppressive and osteogenic properties^17–20^. Calycosin also was demonstrated to ameliorate diabetes-induced cognitive impairments via PI3K/Akt/GSK-3β signaling pathway^21^. Besides, calycosin acts as a Ca^2+^ channel blocker, which may block voltage-dependent Ca^2+^ channel and receptor-operated Ca^2+^channel^22^. In our previous study^23^, we firstly confirmed that pre-treatment with calycosin has a potential neuroprotective effect on ischemia and reperfusion-induced cerebral damage in rats. However, whether calycosin will also provide neuroprotection when given after the onset of stroke and what is the key mechanism underlying its neuroprotective effect still need to be explored. The present study is aimed to investigate the effect of calycosin post-treatment on cerebral ischemic injury and the role of TRPC6-CREB pathway in this phenomenon.

## Results

### Calycosin alleviated MCAO-induced brain injury

**Figure 1:**
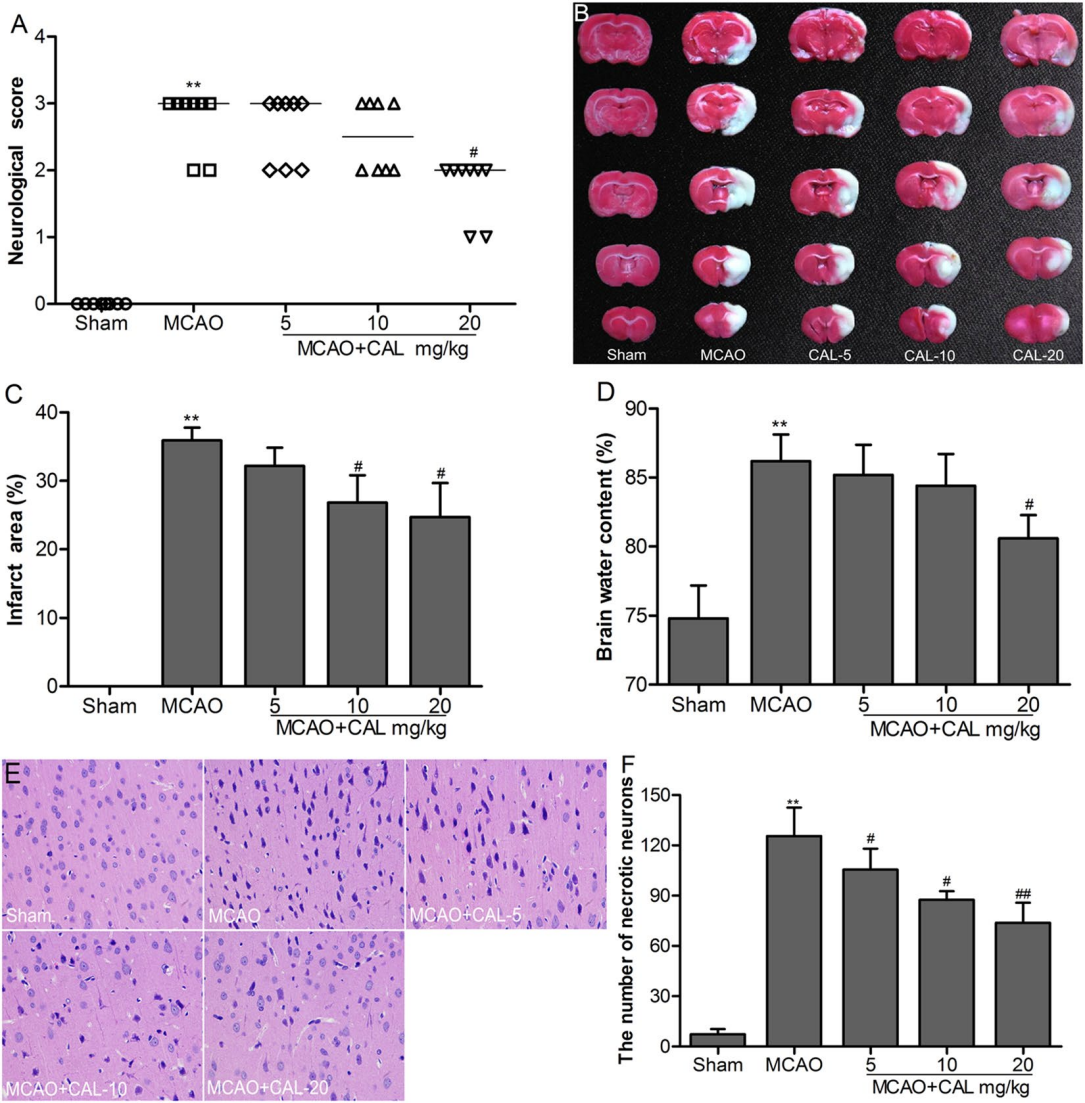
Calycosin (CAL) alleviated MCAO-induced brain injury. (**A**) Scatterplot of neurological score in the sham, MCAO, 5, 10, 20 mg/kg calycosin treatment groups (Data were presented as median, n = 8). (**B**) TTC staining of the cerebral infarct in each group. (**C**) Statistical analysis of the percentage of infarct area in each group (n = 6). (**D**) The percentage of brain water content in each group (n = 6). (**E**) H–E stains of coronal sections from the ischemic penumbral cortex in the sham, MCAO, 5, 10, 20 mg/kg calycosin treatment groups. (**F**) Necrotic neurons were counted in each group (n = 6). All data, except for neurological score, were expressed as mean ± S.D; **P < 0.01 compared with the sham group; ^##^ *P* < 0.01, ^#^ *P* < 0.05 compared with MCAO group.

Neurologic scores showed no significant neurological deficits in sham group, while severe neurological deficits were observed in MCAO group (*P* < 0.01 *vs*. sham group). Post-treatment with 20 mg/kg calycosin significantly improved the neurological deficits (*P* < 0.05 *vs*. MCAO group; Fig. 1A). There was also no detectable cerebral injury in sham group. A large infarct area was observed in MCAO group (*P* < 0.01 *vs*. sham group), whereas treatment with calycosin at doses of 10 and 20 mg/kg significantly reduced the infarct area when compared with the MCAO group (*P* < 0.05; Fig. 1B and C). As well, post-treatment with 20 mg/kg calycosin showed significantly decline in the brain water content when compared with MCAO group (*P* < 0.05; Fig. 1D). Furthermore, the protective effect of calycosin against cerebral ischemic injury was confirmed by histological observation. The results showed that the number of necrotic neurons of the ischemic penumbral cortex in MCAO group was significantly increased at 24 h after reperfusion compared to that of the sham group (*P* < 0.01). After treatment with calycosin, necrotic neurons were markedly reduced (Fig. 1E and F; *P* < 0.05 or *P* < 0.01 *vs*. MCAO group).

### Calycosin reduced ischemia-induced apoptosis

**Figure 2:**
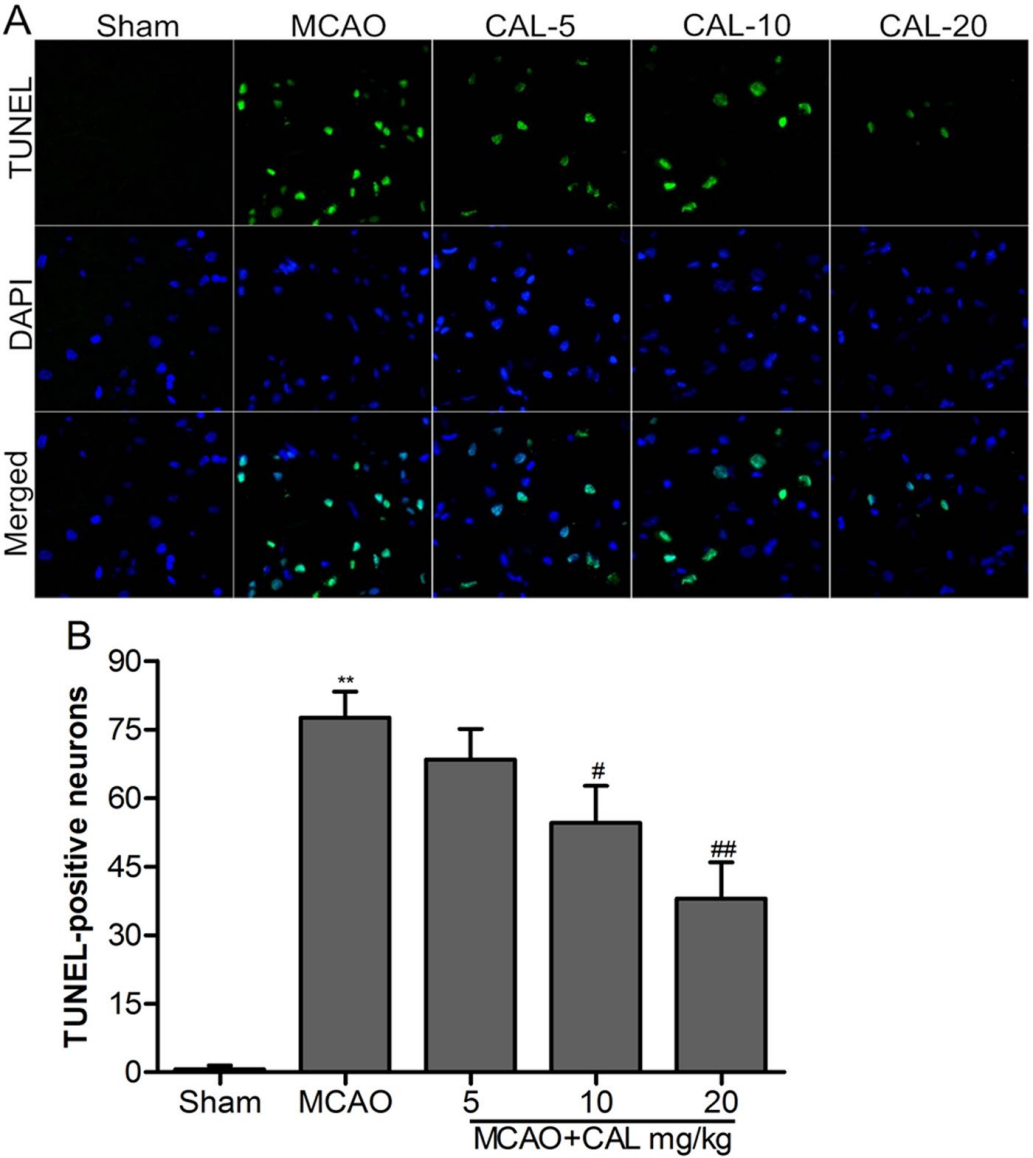
Calycosin (CAL) reduced ischemia-induced apoptosis. (**A**) Representative images of TUNEL staining of the ischemic penumbral cortex in the sham, MCAO, 5, 10, 20 mg/kg calycosin treatment groups. DAPI stain was used to label the nucleus. (**B**) Quantitative analysis of the number of TUNEL-positive neurons in each group (n = 6). Data were presented as mean ± S.D. **P < 0.01 compared with the sham group; ^##^ *P* < 0.01, ^#^ *P* < 0.05 compared with MCAO group.

Cell apoptosis were also assessed by TUNEL staining in the brain. Figure 2A showed TUNEL staining in the ischemic penumbral cortex. The bar graph (Fig. 2B) showed quantitative analysis of the number of TUNEL-positive neurons. The number of TUNEL-positive cells significantly increased after 24 h of reperfusion in MCAO group (*P* < 0.01 *vs*. sham group). The number of TUNEL-positive cells in 10, 20 mg/kg calycosin-treated group were decreased compared to that of the MCAO group (*P* < 0.05 and 0.01, respectively), while decrease of TUNEL positive cells in 5 mg/kg calycosin group did not reach statistical significance.

### Calycosin enhanced the expressions of TRPC6 and P-CREB, and inhibited calpain activation in MCAO rats

**Figure 3:**
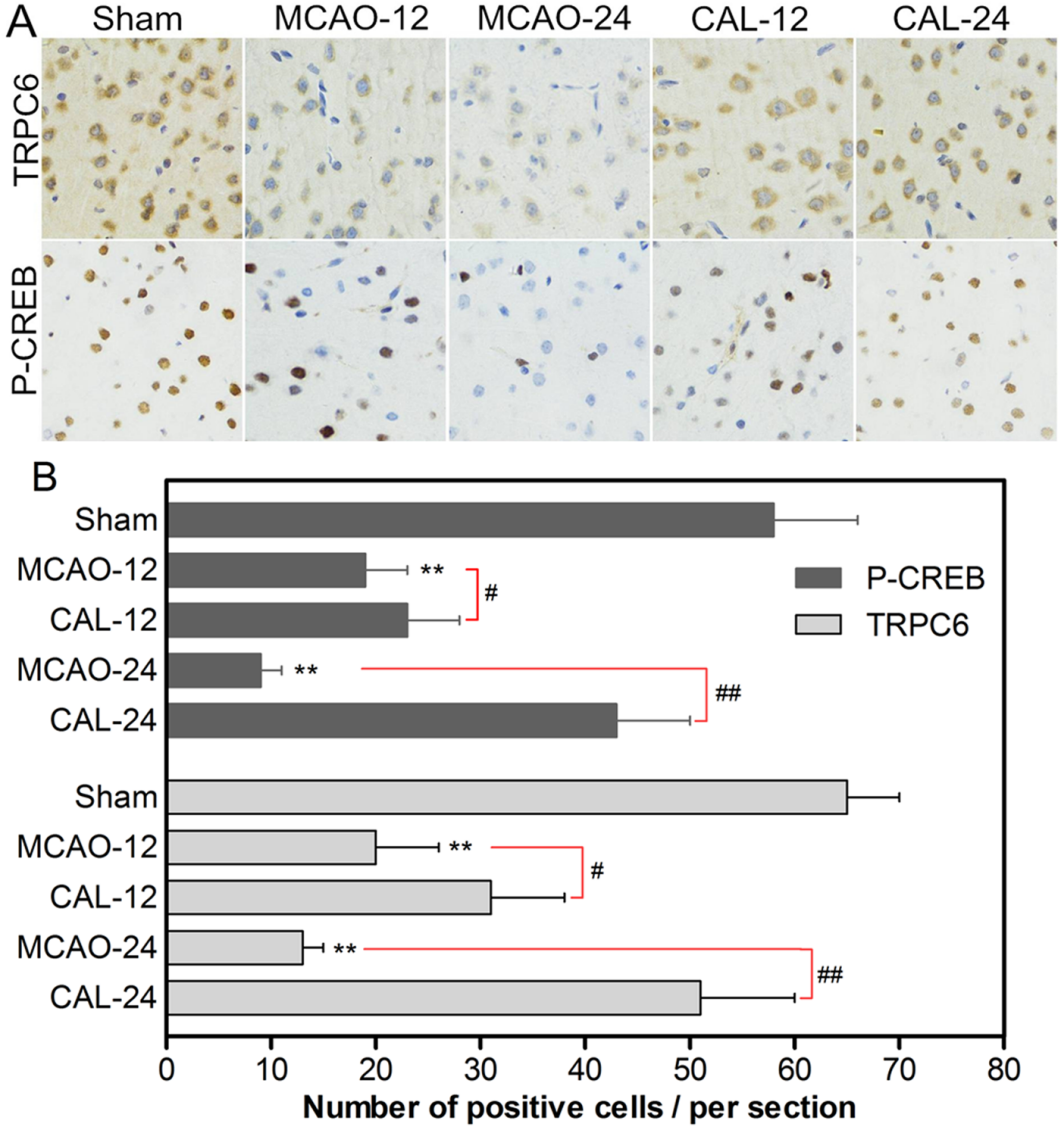
Calycosin (CAL) increased the number of TRPC6 and P-CREB positive cells at 12 and 24 h after reperfusion. (**A**) Representative sections of immunohistochemical staining in the ischemic penumbral cortex in the sham, MCAO and calycosin treatment groups. (**B**) Quantitative analysis of the number of positive immune cells (n = 6). Data were expressed as mean ± S.D; **P < 0.01, compared with the sham group; ^##^ *P* < 0.01, ^#^ *P* < 0.05, compared with MCAO group at the same time point.

**Figure 4:**
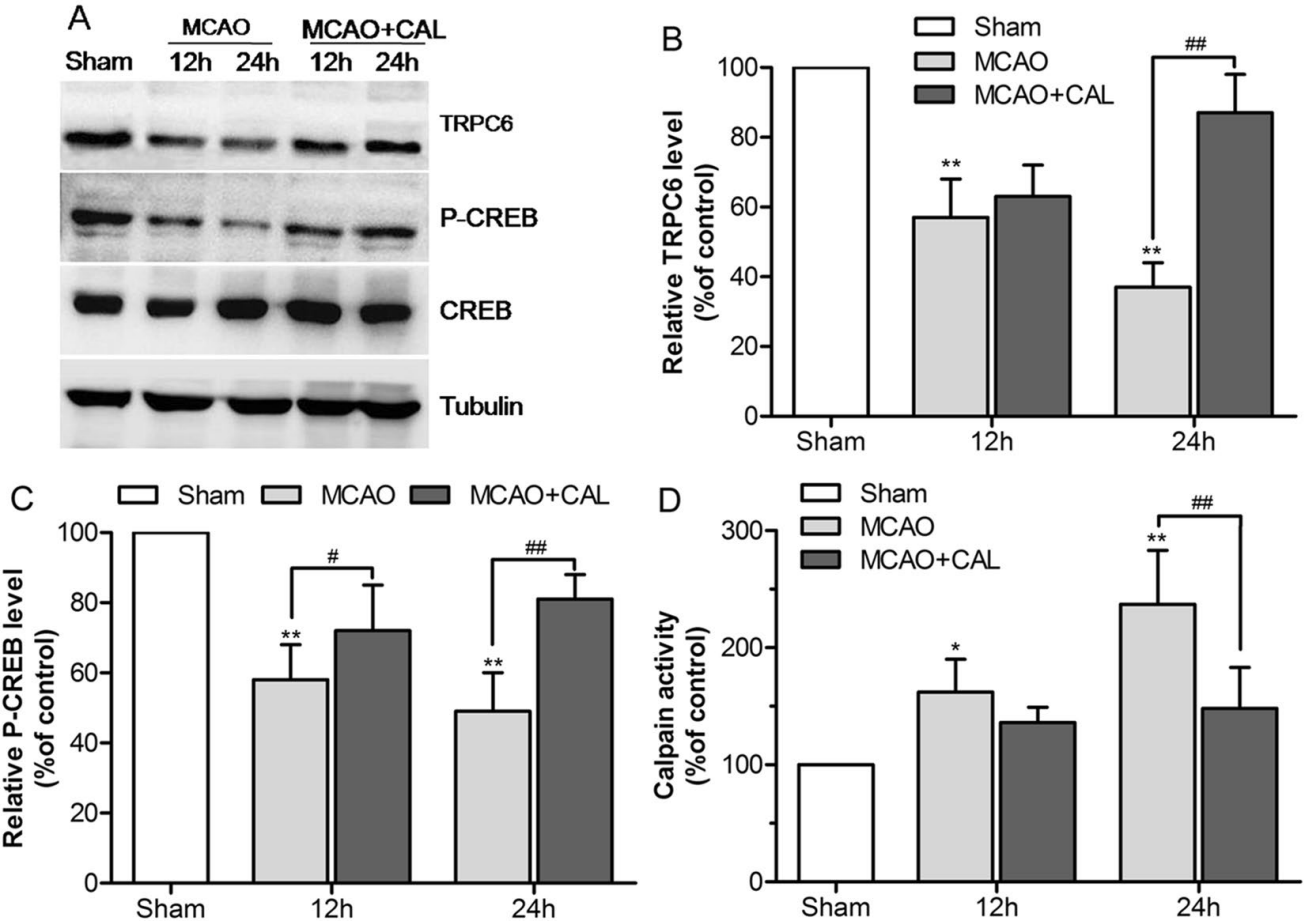
Calycosin (CAL) raised the levels of TRPC6 and P-CREB, and inhibited calpain activation at 12 and 24 h after reperfusion. (**A**) Representative western blot bands of TRPC6, P-CREB, CREB and tubulin are shown in the sham, MCAO and calycosin treatment groups. (**B**,**C**) The quantification analysis of the expression levels of TRPC6 and P-CREB (n = 6). (**D**) Calpain activities in the sham, MCAO and calycosin treatment groups were measured (n = 6). Data were expressed as mean ± S.D; **P < 0.01, *P < 0.05 compared with the sham group; ^##^ *P* < 0.01, ^#^ *P* < 0.05, compared with MCAO group at the same time point.

Immunohistochemical staining of TRPC6 and P-CREB in the ischemic penumbral cortex were shown in Fig. 3A and B. TRPC6 and P-CREB positive staining neurons were reduced in the MCAO group at 12 h and 24 h after reperfusion compared to that of sham group (*P* < 0.01). Treatment with calycosin markedly reversed the reduction of TRPC6 and P-CREB by MCAO injury (*P* < 0.05 or *P* < 0.01 *vs*. that of MCAO group at the same time point, respectively). Western blot results (Fig. 4A) were similar to immunohistochemistry staining. Figure 4B and C showed that the levels of TRPC6 and P-CREB in the MCAO group were markedly decreased at 12 h and 24 h after reperfusion (*P* < 0.01 *vs*. sham group). In calycosin-treated group, the levels of TRPC6 and P-CREB nearly recovered to the normal values at 24 h after reperfusion (*P* < 0.01 *vs*. MCAO group); TRPC6 and P-CREB levels at 24 hours was higher than that at 12 h. As shown in Fig. 4D, calpain activity in the MCAO group was distinctly strengthened at 12 h and 24 h after reperfusion when compared with the sham group (*P* < 0.05 or *P* < 0.01). When MCAO rats were treated with calycosin, calpain activity was significantly weakened at 24 h after reperfusion compared to MCAO group (*P* < 0.01).

### Inhibitor of SKF96365 reversed the neuroprotection effect of calycosin

**Figure 5:**
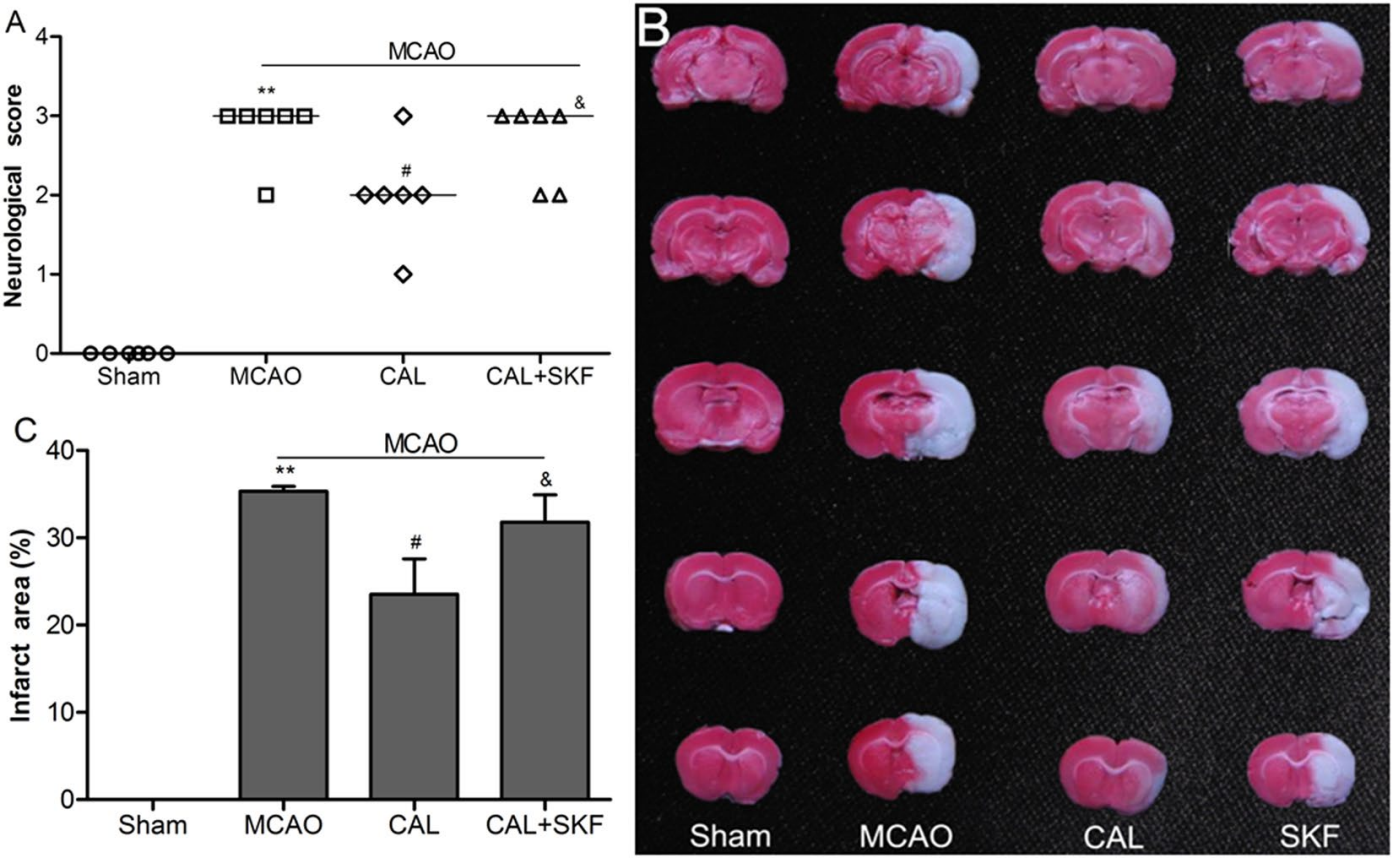
SKF96365 reversed the neuroprotection effect of calycosin. (**A**) Neurological score in sham, MCAO, calycosin (CAL) and calycosin plus SKF96365 (CAL + SKF) groups (Data were presented as median, n = 6). (**B**) TTC staining for the cerebral infarct area in each group. (**C**) Statistical analysis of the percentage of infarct area (n = 6). Data were expressed as mean ± S.D; **P < 0.01, compared with the sham group; ^#^ *P* < 0.05, compared with MCAO group; ^&^ *P* < 0.05, compared with MCAO + CAL group.

To investigate the role of TRPC6 in mediating the neuroprotection of calycosin against MCAO-induced brain injury, we administered rats SKF96365 to inhibit the protein expression of TRPC6. Neurologic scores and infarct area ratio were performed at 24 h after reperfusion. As shown in Fig. 5A, administration of SKF96365 abolished the improvement of neurologic deficits provided by calycosin post-treatment (*P* < 0.05 *vs*. calycosin group). Also, the infarct area ratio in calycosin + SKF96365 group was significant increased compared to calycosin group (*P* < 0.05; Fig. 5B and C). The results indicated that TRPC6 channel may mediate the neuroprotection of calycosin after cerebral ischemia injury.

### Calycosin protected neuron cell against OGD injury

**Figure 6:**
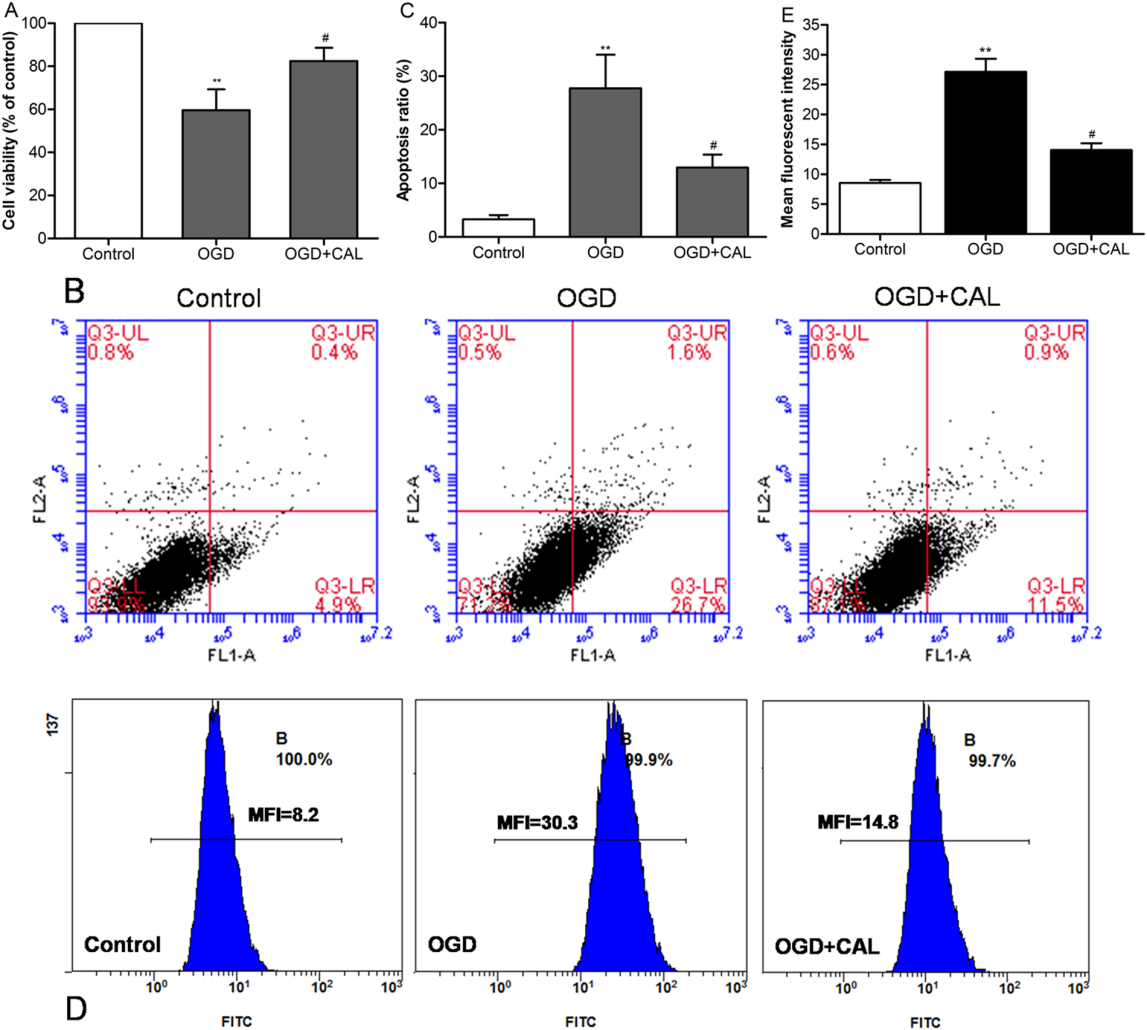
Calycosin (CAL) protected neuron cell against OGD injury. (**A**) Histogram showing cell viability at 24 h after OGD in control, OGD and OGD + calycosin groups. (**B**) Representative pictures of flow cytometry analysis for apoptosis in each group; FL1: FITC, FL2: PI. (**C**) The percentage of apoptotic cells of different groups at 24 h after OGD (n = 6). (**D**) Measurement of the intracellular Ca^2+^ concentration by flow cytometry after incubation in fluo-3/AM. (**E**) The mean fluorescence intensity (MFI) of intracellular Ca^2+^ in different groups. Data were expressed as mean ± S.D; **P < 0.01, compared with the control group; ^#^ *P* < 0.05, compared with OGD group.

We further investigated the neuroprotective effect of calycosin *in vitro* by CCK-8 and flow cytometry analysis. The result of cell viability showed that calycosin alleviated neuron cell death induced by 2 h of OGD injury (*P* < 0.05 *vs*.OGD group; Fig. 6A). Quantitative analysis of apoptosis revealed that 4 h of calycosin treatment given prior to OGD exposure significantly decreased cell apoptosis after OGD injury, compared to that of OGD group alone (*P* < 0.05; Fig. 6B and C). Furthermore, we also found that the concentration of intracellular Ca^2+^ rapidly increased after OGD injury (*P* < 0.01 *vs*. control group), while treatment with calycosin significantly suppressed the elevation of intracellular Ca^2+^ when compared to OGD group (*P* < 0.05; Fig. 6D and E).

### Calycosin altered the protein and mRNA expression levels of TRPC6 and P-CREB in cells

**Figure 7:**
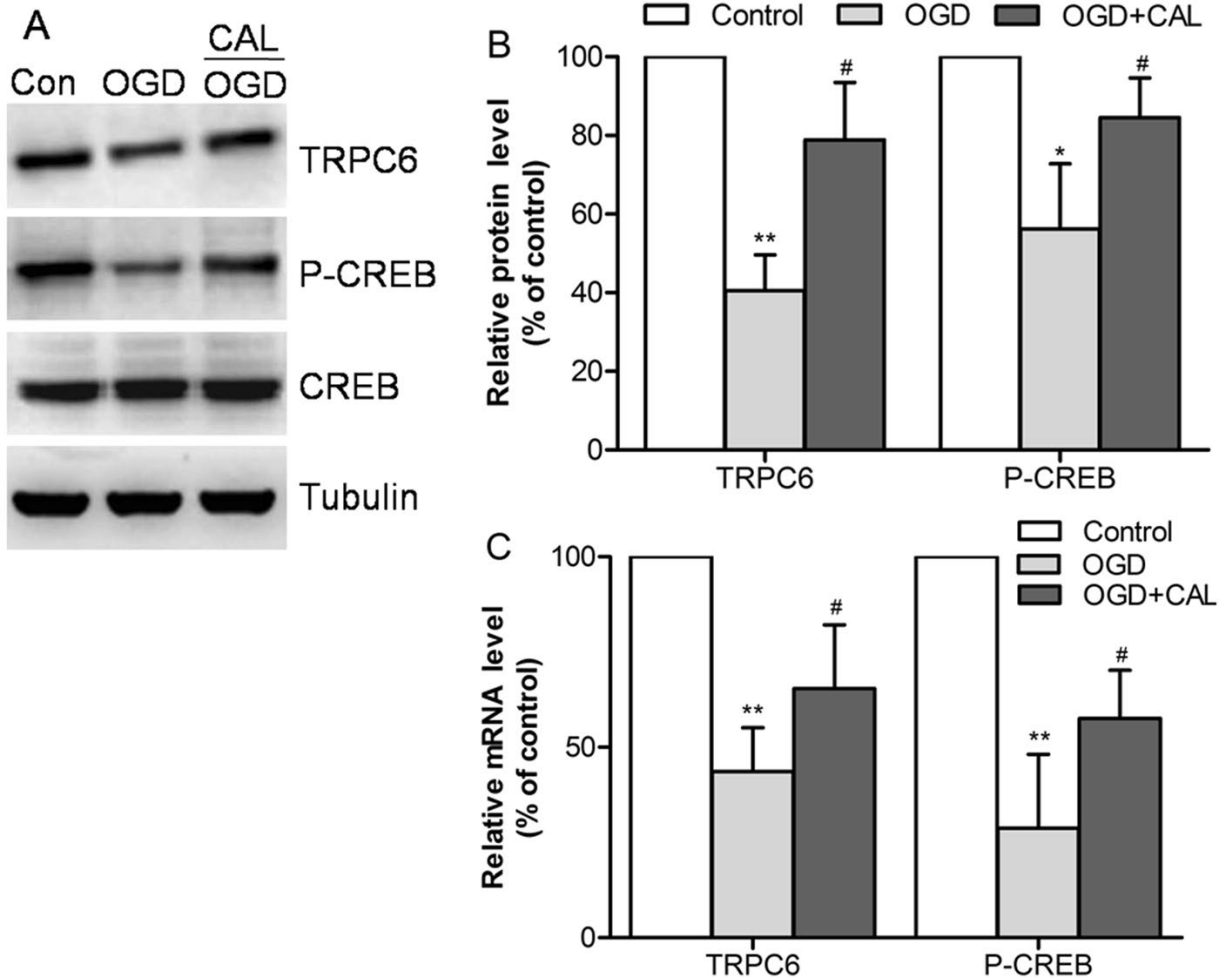
Calycosin (CAL) altered the protein and mRNA levels of TRPC6 and P-CREB at 24 h after OGD. (**A**) The bands of TRPC6, P-CREB, CREB and tubulin in control, OGD and OGD + calycosin groups. (**B**) Quantification of TRPC6 and P-CREB levels in each group (n = 6). (**C**) The mRNA levels were determined by RT-PCR (n = 6). Data were expressed as mean ± S.D; *P < 0.05, **P < 0.01, compared with the corresponding control group (con); ^#^ *P* < 0.05, compared with the corresponding OGD group.

As shown in Fig. 7A and B, the results suggested that the incubation with OGD decreased the proteins levels of TRPC6 and P-CREB in primary cultured neuron (*P* < 0.05 or *P* < 0.01 *vs*. control group). In contrast, treatment with calycosin significantly prevented the decrease of TRPC6 and P-CREB levels brought by OGD (*P* < 0.05 *vs*. OGD group). Similar changes of mRNAs were shown in Fig. 7C.

### The neuroprotection of calycosin involved the TRPC6/CREB pathway

**Figure 8:**
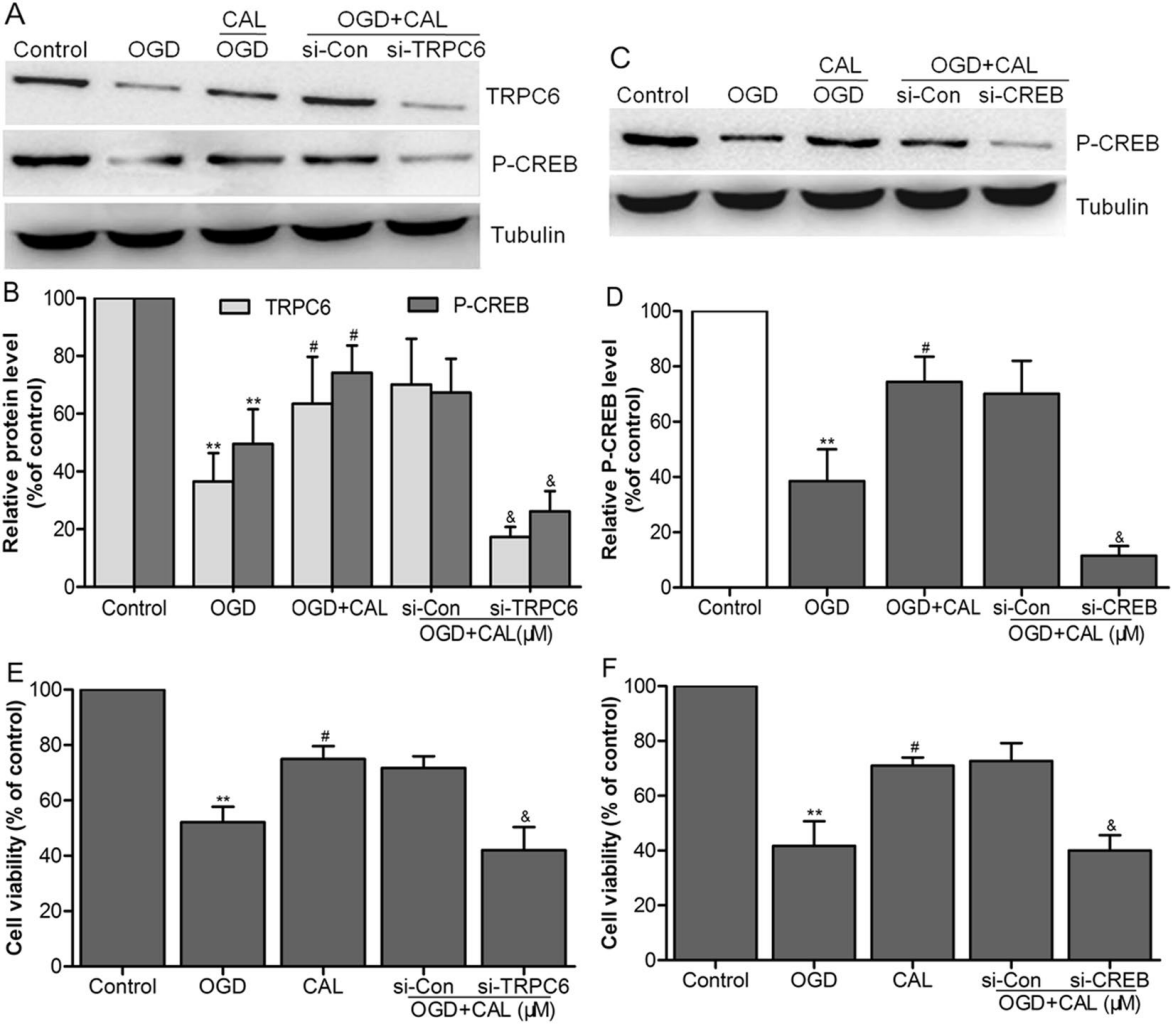
The neuroprotection of calycosin involved the TRPC6/CREB pathway. (**A**,**C**) Representative western blot bands showing the expression levels of TRPC6 and P-CREB in each group when cells were transfected with TRPC6 siRNA (si-TRPC6) or CREB siRNA (si-CREB) and control siRNA (si-Con) for 48 h. (**B**,**D**) The quantification of TRPC6 and P-CREB levels in each group. (**E**,**F**) The percentage of cell viabilities in each group was measured by CCK-8 kit. Data were presented as mean ± S.D. (n = 6). **P < 0.01 compared with the control group; ^#^ *P* < 0.05 compared with OGD group; ^&^ *P* < 0.05 compared with control siRNA (si-Con) group.

To further verification of the role of TRPC6/CREB pathway in mediating the protective effect of calycosin against OGD-induced injury, we used small interference RNAs of TRPC6, CREB or scramble negative control RNAs (si-Control) in primary cultured neurons for 48 h and then evaluated the expressions of TRPC6 and CREB by western blot. We found that transfection with si-TRPC6 significantly decreased the expression of TRPC6 and P-CREB in calycosin treated cells after OGD compared to that of cells transfected with si-Control (*P* < 0.05; Fig. 8A and B). Besides, the expression of P-CREB obviously decreased after si-CREB transfection (*P* < 0.05, MCAO + calycosin + si-CREB *vs*. MCAO + calycosin + si-Control group; Fig. 8C and D). With the inhibition of TRPC6 and CREB expression by siRNAs, cell viability preservation provided by calycosin was also markedly reversed after OGD (Fig. 8E and F).

## Discussion

Stroke is an enormous public health problem with an imperative need for more effective therapies. Calycosin, an isoflavonoid phytoestrogen isolated from *Radix Astragali Mongolici*, was firstly reported to be neuroprotective in rats by our group. But its molecule mechanisms underlying the neuroprotective effect have not been thoroughly investigated. In the present study, we found that: (1) Cerebral ischemia obviously led to intracellular Ca^2+^ overload, activated calpain, and decreased the expressions of TRPC6 and P-CREB. (2) Treatment with calycosin protected the brain against ischemic injury through up-regulating TRPC6 and P-CREB expression and inhibiting calpain activation in both in *vitro* and in *vivo* model of ischemia. (3) The neuroprotective effect of calycosin was diminished by the inhibition or knockdown of TRPC6 and CREB. These findings indicated that the potential neuroprotection mechanism of calycosin was involved with Ca^2+^ overload-related TRPC6 channel.

An important mechanism of neuronal cell death following brain ischemia is the intracellular Ca^2+^ overload. According to calcium overload hypothesis^24, 25^, the accumulated high Ca^2+^ levels lead to mitochondrial dysfunction and generation of reactive oxygen species (ROS), and over activate Ca^2+^-dependent enzymes such as proteases, phospholipases and endonucleases. The breakdown of proteins, lipids and nucleic acids by these enzymes contribute to cell death. Calpain is a Ca^2+^-dependent protease that belongs to the member of papaya proteinase family of the cysteine proteinase superfamily, which is ubiquitously expressed in all mammalian tissues and cell types^26, 27^. Under the normal physiologic condition, calpain activity is likely to be reversibly stimulated by transient elevation of localized cytosolic Ca^2+^. And calpain activity is also tightly regulated by its specific endogenous inhibitor-calpastatin^28^. During brain ischemia, the rapid elevation of cytosolic Ca^2+^ overwhelms its endogenous counteracting regulatory system and results in pathologic increase of calpain activity^29^. Many previous studies have shown that calpain activity is increased by focal cerebral ischemia, and calpain inhibitors provide varying degrees of neuroprotection in animal models^30–32^. For example, Neumar *et al*. demonstrated that calpain is activated in the hippocampus, cortex, and striatum with an initial increase at 1 h followed by a more prominent secondary increase at 36 h after cerebral ischemia^33^. Jiang *et al*. reported the time course of μ-calpain activation in hypoxic-ischemic neonatal rat brain. And the activation of μ-calpain was increased significantly and reached a maximum at 24 h in cortex and at 12 h in hippocampus^34^. The two major isoforms of calpain found in brain tissue are known as μ-calpain (calpain-1) and m-calpain (calpain-2), but their abundant expression differs among cell types^35, 36^. μ-calpain is mainly expressed in neurons while m-calpain is more abundant in glial cells. In addition, μ-calpain activation only requires the micromolar level of Ca^2+^, whereas m-calpain activation needs millimolar level of Ca^2+^. Calpain is a specific calcium-dependent protease, while calycosin had been confirmed to inhibit the calcium channel^22^. In the current study, we further demonstrated an over-activation of calpain in the ischemic penumbra cortex following MCAO in rats demonstrated by activity assay, whereas treatment with calycosin significantly inhibited calpain over-activation.

The activation of calpain is involved in ischemic brain injury through various ways, such as hydrolysis of cytoskeletal and membrane proteins, activation different enzymes, etc., which ultimately lead to cell death^37–39^. Transient receptor potential channels (TRPCs) are Ca^2+^-permeable, nonselective cation channels with several physiological functions, including growth cone guidance^40^, neurite outgrowth^41^, muscle cell proliferation^42^, fear memory^43^, and neuronal survival etc.^44^. The TRPC subfamily with 7 subunits can be divided into 4 subgroups based on their sequence similarity: TRPC1, TRPC2, TRPC3/6/7, and TRPC4/5, of which TRPC6 is widely expressed in neuron. Calpain-mediated proteolysis of TRPC6 contributes to ischemic neuronal cell death^45^, and suppression of calpain activity inhibits TRPC6 degradation and could prevent ischemic brain damage^46^. Therefore, TRPC6 have a critical role in promoting neuronal survival against focal cerebral ischemia. Up-regulation of TRPC6 channel also increased markedly its downstream cAMP response element-binding protein (CREB) phosphorylation and enhanced CREB-dependent transcription^47^. CREB, belonging to the family of leucine zipper transcription factors, is critical to induce its effects at phosphorylation of a serine residue (S133) in its kinase-inducible domain^48^. Phosphorylation of CREB can be accomplished by a number of upstream signaling cascades. The CREB activation was a critical step in neuroprotection against ischemic injury^49, 50^. In nervous system, influx of Ca^2+^ through TRPC6 channel result in Ca^2+^-dependent activation of CaMKIV, which in turn activate CREB transcriptional pathways to promote neuronal survival^44^. In the present study, our data showed that the expression of TRPC6 and P-CREB in the MCAO group significantly decreased at 12–24 h after reperfusion. Calycosin post-treatment significantly inhibited the degradation TRPC6 and P-CREB after MCAO as proved by immunohistochemistry and western blot analysis. *In vitro* studies using primary cultured neurons showed similar results. In addition, we used a TRPC inhibitor, SKF96365 to investigate whether TRPC6 is critical in calycosin-induced protection against cerebral ischemia. Our results demonstrated that administration of SKF96365 at 20 min before initiation of MCAO significantly reversed calycosin-induced brain protection after MCAO, suggesting that the neuroprotective effect of calycosin depends on TRPC6 up-regulation. To provide further evidence for the critical role of TRPC6-CREB pathway in calycosin-induced neuroprotection, we used RNA interference technique to directly knockdown the expression of TRPC6 or CREB. The results confirmed that knockdown of TRPC6 and CREB abolished the neuroprotection provided by calycosin *in vitro*. Taken together, our studies demonstrated that calycosin blocked calpain-mediated degradation of TRPC6 channel that modulate intracellular Ca^2+^ levels to stimulate CREB phosphorylation, resulting in neuroprotection against cerebral ischemia in both *in vivo* and *in vitro* models.

In conclusion, our study suggested that post-treatment with calycosin significantly attenuated cerebral ischemic damage. And calycosin exerted its neuroprotective effects by inhibiting calpain activity and increasing the expressions of TRPC6 and P-CREB. These results provide a better understanding of the molecular mechanisms underlying the neuroprotective effect of calycosin and may provide new insight into better design of neuroprotective agents against ischemia stroke.

## Materials and Methods

### Drugs and Reagents

Calycosin (molecular weight 284.26, CAS 20575-57-9, purity >99%) was supplied by the weikeqi biotech co., Ltd (Chengdu, China). Fetal bovine serum (FBS), Dulbecco’s modified Eagle’s medium (DMEM)/Ham’s F12, B27 and L-glutamine supplements were purchased from Gibco Life Technologies (Grand Island, NY, USA). 2, 3, 5-triphenyltetrazolium chloride (TTC) and Dimethyl Sulfoxide (DMSO) were obtained from Sigma-Aldrich (St. Louis, MO). The protein extraction kit and BCA protein assay kit were purchased from Nanjing Jiancheng Bioengineering Institute (Nanjing, China). Total RNA extraction with TRIzol, Lipofectamine 3000 transfection reagents and Fluo-3/AM were purchased from Invitrogen Life Technologies (Carlsbad, CA, USA). The antibodies of TRPC6 and β-tubulin, calpain activity assay kit and the inhibitor of SKF96365 were purchased from Abcam plc (Abcam, Cambridge, UK). Anti-CREB and anti-P-CREB (Ser133) antibodies were products of Cell Signaling Technology, Inc (CST, Danvers, MA, USA). Terminal deoxynucleotidyl transferase dUTP nick end labeling (TUNEL) assay kit, Cell Counting Kit-8 (CCK-8) and Annexin V-FITC apoptosis detection kit were purchased from Beyotime Institute of Biotechnology (Nantong, China).

### Experimental Protocol

#### *In vivo* studies

Experiment 1: Effect of calycosin post-treatment on middle cerebral artery occlusion (MCAO) -induced brain injury. Rats were randomly assigned to five groups: sham-operated group (sham), MCAO group, and three MCAO + calycosin (5, 10, 20 mg/kg) treatment groups. Claycosin was dissolved in 100 mM DMSO solvent. At the onset of reperfusion, rats were given solvent or the different doses of calycosin by intraperitoneal injection. After 24 h of reperfusion, neurologic score, infarct area ratio and brain water content were evaluated by a researcher blinded to the grouping. Histological observation and apoptosis were performed by hematoxylin-eosin (H-E) and TUNEL staining, respectively.

Experiment 2: Examination of calpain activity, TRPC6 and P-CREB expressions in the MCAO rat following post-treatment with calycosin. According the results from experiment 1, 20 mg/kg calycosin was used in the treatment group. Rats were randomly allocated to three groups: sham, MCAO, and MCAO + calycosin groups. At the onset of reperfusion, rats were given solvent or 20 mg/kg calycosin by intraperitoneal injection. Immunohistochemistry and western blot analysis for TRPC6 and P-CREB, and calpain activity assay were performed at 12 h and 24 h after reperfusion.

Experiment 3: Exploration of the role of TRPC6 in mediating the protective effect of calycosin against MCAO-induced brain injury. Rats were randomly divided into four groups: sham, MCAO, MCAO + calycosin, MCAO + calycosin + SKF96365. 5 μl SKF96365 (TRPC inhibitor, dissolved in artificial cerebrospinal fluid, 1 mM) was administered by intracerebroventricular injection (coordinates: 0.8 mm posterior to bregma, 1.5 mm lateral to sagittal suture and 3.6 mm ventral from the surface of brain.) at 20 min before MCAO. After 24 h of reperfusion, neurologic score and infarct area ratio were evaluated according to the method described experiment 1.

### Animals and middle cerebral artery occlusion (MCAO) model

Adult male Sprague-Dawley rats (220~240 g) were obtained from the Experimental Animal Center of the Fourth Military Medical University. Experimental protocols were approved by the Ethics Committee for Animal Experimentation of the Fourth Military Medical University (Xi’an, China) and in accordance with the National Institutes of Health Guide for the Care and Use of Laboratory Animals. MCAO was induced using an intraluminal monofilament occlusion as previously described^51^. In brief, rats were anesthetized with 10% chloral hydrate (3 mL/kg, I.P) and placed in supine position. Body temperature was regulated a 37 °C by homoiothermy bench. Then the right common carotid artery, the external carotid artery (ECA) and the internal carotid artery (ICA) were exposed and carefully isolated. A 3-0 monofilament nylon suture (Beijing Cinontech Co. Ltd, China) was inserted through a small incision on the ECA and forwarded to the root of the right middle cerebral artery (MCA), where the rounded tip would block blood flow to MCA supplied regions. After 2 h of MCAO, the suture was removed to restore blood flow. Sham-operated rats underwent the same surgical procedure except that the suture was not inserted into the ICA. To monitor occlusion and reperfusion, the regional cerebral blood flow (rCBF) was monitored by laser Doppler flowmetry (PeriFluxsystem 5000; Perimed AB, Stockholm, Sweden) positioned at 1 mm posterior and 5 mm lateral to bregma. An 80% drop and 70% recovery in rCBF was considered as a successful MCAO operation.

### Neurologic deficits score

After 24 h of reperfusion, neurological deficits were evaluated by a blinded observer with a 5-point scale scoring system as described previously^52^. 0 = no deficit; 1 = failure to extend left forepaw fully; 2 = circling to the left; 3 = falling to the left; 4 = no spontaneous walking with a depressed level of consciousness.

### Measurement of infarct area and brain water content

Infarct areas were measured as described previously^53^. After neurological score, the brains were sliced into uniform coronal sections of 2 mm thickness each and slices were stained using 2% TTC at 37 °C for 5~10 min. The TTC-stained sections were photographed with a digital camera and the infarct areas of each section were measured using an image analysis system. To exclude possible confounding effects of brain edema, an indirect method was used to calculate the infarct areas. Infarct areas were expressed as percentages of contralateral hemispheric areas.

Subsequently, all of tissues including infarct section and non-infarct sections were weighed immediately to obtain the wet weight. Then these tissues were dried in a desiccating oven at 110 °C for 24 h and weighed again to obtain the dry weight. Brain water content was calculated as follows:

$${\rm{brain}}\,{\rm{water}}\,\mathrm{content}\,( \% )=({\rm{wet}}\,{\rm{weight}}-{\rm{dry}}\,{\rm{weight}})/{\rm{wet}}\,{\rm{weight}}\times 100 \% .$$

### Tissue specimen preparation

For H-E staining, TUNEL and immunochemistry analysis, rats were deeply at 24 h after reperfusion and infused with 0.1 M phosphate buffered saline (PBS) to wash off the blood, followed by freshly prepared 4% (w/v) paraformaldehyde in 0.1 M PBS (pH = 7.4). Then brain was removed and post-fixed in 4% paraformaldehyde for 24 h. Brain blocks were embedded in paraffin after dehydration and cut into 5 µm coronal sections. Sections were used for H-E, TUNEL and immunochemistry staining. For western blot analysis, rat brains were removed and the ischemic penumbral cortices were quickly dissected on ice. Samples were stored at 80 °C until ready for use.

### Histological observation and TUNEL assay

Standard H-E staining was performed for histological observation. The number of necrotic neurons in the ischemic penumbral cortex was counted in a blind manner by light microscopy at ×400 magnifications (IX71; Olympus Corporation, Tokyo, Japan) in 3 different slides at bregma ±2.5 mm for each animal. TUNEL assay was also performed in 3 slices per animal, according to the manufacture’s instruction of One Step TUNEL Apoptosis Assay Kit. Briefly, deparaffinized and rehydrated sections were treated with proteinase K for 20 min and subsequently incubated with FITC-labelled TUNEL staining for 1 h at 37 °C. The TUNEL-positive cells were imaged under a fluorescent microscope (IX71; Olympus Corporation, Tokyo, Japan) and quantified as the number of green spots in each observation field (×400). Data from 6 animals in each group were averaged.

### Immunohistochemistry analysis

After deparaffinization and antigen retrieval, sections were first incubated with normal goat serum to inhibit nonspecific binding of the antibodies. Then sections were incubated with the primary antibodies of TRPC6 and P-CREB at 4 °C overnight, followed by incubation with a biotinylated secondary antibody for 1 h then HRP-streptavidin for 30 minutes at room temperature. Then sections were developed with diaminobenzidine for 5 min at room temperature. Finally, sections were counterstained with hematoxylin and covered for observation under light microscope (IX71; Olympus Corporation, Tokyo, Japan).

### Calpain activity assay

Calpain activity in the ischemic penumbral cortices was determined using a calpain activity assay kit (Abcam) according to the manufacturer’s protocol. Briefly, cortices were homogenized in lysis buffer at 4 °C. The supernatant were then incubated with substrate (Ac-LLY-AFC) and reaction buffer for 1 h at 37 °C in the dark. Upon cleavage of substrate, the fluorogenic portion (AFC) releases yellow-green fluorescence at a wavelength of 505 nm following excitation at 400 nm. Fluorescence emission was measured in a standard fluorimeter.

#### *In vitro* studies

Experiment 1: Identification of the expressions of TRPC6 and P-CREB in neuron cells after oxygen-glucose deprivation (OGD) and treatment with calycosin. Primary cultured neuron cells were divided into three groups: control, OGD and OGD + calycosin groups. Based our preliminary *in vitro* research, the dosage of calycosin was 60 μM in the present study. Cells were harvested at 24 h after OGD to evaluate cell viability, apoptosis, Ca^2+^ concentration, and mRNA as well as protein levels of TRPC6 and P-CREB.

Experiment 2: Verification of the role of TRPC6/CREB pathway in mediating the protective effect of calycosin against OGD-induced injury. Small interference RNA (siRNA) was used. Cells were assigned to five groups: control, OGD, OGD + calycosin, siControl + OGD + calycosin, siRNA + OGD + calycosin. Cell viability was evaluated at 24 h of after OGD.

### Cell culture and oxygen glucose deprivation (OGD)

Cerebral cortices were isolated from pregnant SD rats at embryonic day 17 as described previously^54^. Rats were obtained from the Experimental Animal Center of the Fourth Military Medical University. In brief, the pregnant SD rats were anesthetized and the embryos were dissected from uterine tissue. Then the cerebral cortices from the embryos were digested with 2 mg/ml fresh papain for 20 min at 37 °C. After terminating the digestion, neurons were seeded on poly-L-lysine-coated culture dishes and maintained in DMEM/F12 medium supplemented with 10% fetal bovine serum, 100 U/mL penicillin and 100 mg/mL streptomycin at 37 °C in a humidified incubator with 5% CO_2_. After 4 h, the medium was replaced with neurobasal medium containing 2% B27 and 1 mmol/L glutamine. After 3 days, 5 mM cytosine arabinofuranoside was added to inhibit grow of non-neuronal cells. Culture medium was replaced once every 2 days. Before exposure to OGD, the cultured neurons were pretreated with 60 μM calycosin for 4 h under conditions. To establish OGD model *in vitro*, the cells were incubated with glucose-free Earle’s balanced salt solution (EBSS) and placed in a hypoxic chamber (95% N_2_ + 5% CO_2_) at 37 °C for 2 h. Control neuron cultures were placed in EBSS containing 25 mM glucose and incubated under normal culture conditions for the same period. OGD was terminated by removing cultures from the chamber and changing the media back to the normal culture medium.

### Cell viability assay

Cell viability was determined using a nonradioactive cell counting kit (CCK-8) assay. Briefly, cells were seeded in 96-well plates with 100 μL medium. CCK-8 solution added to the cell culture medium to a final concentration of 5 μL/100 μL, and incubated for an additional 2 h at 37 °C. Subsequently, the absorbance of samples was measured on a microplate reader (Multiskan GO, Thermo scientific, Waltham, MA, USA) at a wave length of 450 nm. Cell viability was expressed as a percentage with the control group as 100%.

### Flow cytometry analysis for apoptosis

Cell apoptosis was performed by flow cytometry. Briefly, cells were harvested and washed twice with PBS after 24 h of OGD. The cells were resuspended with 500 μL binding buffer and incubated for 15 min in the dark with 5 μL Annexin V-FITC and 5 μL propidium iodide. Apoptosis was quantified by flow cytometer (Accuri C6, BD Biosciences, San Jose, CA). The apoptosis percentage was summed up from early apoptosis (Annexin V^+^/PI^−^) and late apoptosis (Annexin V^+^/PI^+^). Experiments were repeated three times to ensure reproducibility.

### Measurement of Ca^2+^ concentration

Intracellular Ca^2+^ concentrations were measured by flow cytometry using a fluorescent dye as described previously^55^. Briefly, after removing culture medium, primary cultured neuron cells were loaded with 5 μM Fluo3-AM and 0.04% Pluronic F-127 in Hanks’ balanced salt solution (HBSS) (NaCl 150 mM, KCl 5.4 mM, CaCl_2_ 2 mM, MgCl_2_ 1 mM, glucose 10 mM and HEPES 10 mM, pH = 7.4) for 40 min at 37 °C. The cells were washed twice and resuspended in fresh HBSS solution following incubated at 37 °C for additional 10 min. Mean fluorescence intensity (MFI) of 10,000 cells in each group was analyzed using a Beckman Coulter EPICS XL-MCL flow cytometer (Beckmam Coulter, USA) at the excitation wavelength of 488 nm. Fluorescence intensity can be used to indirectly represent the intracellular Ca^2+^ concentrations.

### RT-PCR

Total RNA was extracted from primary cultured neuron with Trizol reagent according to the manufacturer’s instructions. Total RNA of 1.0 μg was reverse transcribed to complementary DNA (cDNA) using oligo-dT primer and superscript reverse transcriptase. cDNA was amplified in 20 μL reaction volume containing 2 μL of cDNA, 10 μL SYBR Green Supermix and 250 nM of genes specific forward and reverse primers. The cycling parameters were 5 min at 95 °C, 30 cycles at 95 °C for 30 s, 57 °C for 30 s and 72 °C for 30 s. Quantitative real-time PCR (qRT-PCR) was performed using an iCycler (BioRad, Hercules, CA, USA) and the data were analyzed using Bio-Rad CFX Manager software (version 2.0). The expression levels of the genes were normalized to β-tubulin.

### Transient transfection of small RNA interference

The TRPC6, CREB specific small interfering RNAs (siRNAs) and the corresponding non-targeting scramble control siRNA were designed and synthesized by RiboBio Co., Ltd (Guangzhou, China). Primary cultured neurons were transiently transfected with target-specific siRNA or control siRNA by Lipofectamine 3000 regent according to the manufacturer’s protocol. The efficiency of RNA interference was evaluated by Western blotting. Following 48 h transfection, cells were then treated with calycosin and subjected to 2 h OGD challenge followed by reoxygenation for 24 h. Cell samples were collected for CCK-8 assay.

### Western blot analysis

Total protein extracts from the ischemic penumbral cortex or primary cultured neuron were determined using the BCA protein assay kit according to the manufacturer’s instructions. Aliquots of each extract were separated by 10% sodium dodecyl sulfate-polyacrylamide gel electrophoresis (SDS-PAGE) under reducing conditions and then transferred to a PVDF membrane. The membrane was blocked with Odyssey blocking buffer for 3 h at room temperature. Membrane was incubated with anti-TRPC6, anti-P-CREB, and anti-CREB and β-tubulin antibodies overnight at 4 °C. After washed with TBS for 10 min × 3 times, the membranes were then incubated with HRP-conjugated secondary antibody for 2 h at 37 °C. The blots were visualized using the enhanced chemiluminescence method (ECL Kit; Pierce, Rockford, IL, USA), and the bands were scanned and analyzed with Quantity One image analysis software (Bio-Rad Laboratories).

### Statistical analysis

The statistical analyses were performed using SPSS 18.0 for Windows. All data, except for neurologic score, were expressed as mean ± standard deviation (S.D.). One-way analysis of variance (ANOVA) followed by Tukey’s multiple-comparison test was performed for statistical comparison of several groups. Unpaired Student’s t-test was used for comparison of two groups. Neurologic scores were expressed as median (range) and were compared using a nonparametric method (Kruskal-Wallis test) followed by the Mann–Whitney U statistic with Bonferroni correction. A value of *P* < 0.05 was considered as statistically significant.
